# Association of systemic immune-inflammation index (SII) and aggregate index of systemic inflammation (AISI) with thyroid nodules in patients with type 2 diabetes mellitus: a retrospective study

**DOI:** 10.1186/s12902-023-01509-w

**Published:** 2023-11-20

**Authors:** Can Cao, Chunyao Li, Xiaoting Li, Weiwei Sun, Yaoxian Wang

**Affiliations:** Department of Nephrology and Endocrinology, Dongzhimen Hospital, Beijing University of Chinese Medicine, Beijing, 100700 China

**Keywords:** Thyroid nodules, Type 2 Diabetes Mellitus, Systemic Immune-inflammation index, Aggregate index of systemic inflammation

## Abstract

**Objective:**

This retrospective study aimed to investigate the association between TNs and the systemic immune-inflammation index (SII) and the aggregate index of systemic inflammation (AISI) in patients with T2DM.

**Methods:**

A total of 370 T2DM patients, who were admitted to Dongzhimen Hospital between January 2020 and March 2023, were included in this retrospective study. Binary logistic regression models with multivariable adjustment were employed to assess the relationship between SII, AISI quartiles, and TNs. Furthermore, receiver operating characteristic (ROC) curve analysis was performed to assess the diagnostic accuracy of SII and AISI in identifying T2DM patients with TNs.

**Results:**

Age, diabetes duration, diabetic nephropathy (DN), SII, and AISI demonstrated significant positive associations with TNs. Compared to the first quartile of SII, the second, third, and fourth quartiles showed increased risks of TNs with hazard ratios (HRs) of 1.578 (0.883–2.820), 2.279 (1.257–4.131), and 3.626 (1.931–6.810), respectively (P < 0.001). Similar results were observed for AISI and TNs. ROC curve analysis revealed that SII and AISI exhibited a high discriminatory capability for identifying TNs in the overall and male participant group, whereas the significance among females was not discernible.

**Conclusions:**

This study provides evidence that SII and AISI are independent risk factors for TNs, suggesting that elevated SII and AISI levels may contribute to the development of TNs in patients with T2DM particularly among male individuals.

## Introduction

Type 2 diabetes (T2DM) and thyroid diseases are prevalent endocrine disorders with an evident interconnection. Previous studies have revealed a considerably higher prevalence of thyroid nodules (TNs) among T2DM patients compared to individuals without T2DM. Specifically, the prevalence of TNs was found to be 60% in T2DM patients, significantly higher than the rate of 43% observed in those with normal glucose levels [1]. Consequently, it becomes imperative to identify TNs in patients diagnosed with T2DM.

The development of TNs in T2DM is attributed not only to genetic factors, iodine deficiency, and thyroid hormone imbalances [2], but also to insulin resistance and chronic inflammatory states [3, 4]. Recently, systemic immune-inflammation index (SII) and aggregate index of systemic inflammation (AISI) have emerged as novel inflammatory markers. These indices comprehensively integrate various inflammatory cell types present within the body, offering a more comprehensive evaluation of the overall inflammatory status. Extensive research has explored their utility not only in the context of malignancies and other diseases, but also in the field of endocrinology. Notably, SII and AISI have been found to be closely associated with the onset and progression of T2DM [5–8]. In individuals with T2DM, higher levels of inflammation, as indicated by elevated SII and AISI, have been observed. We postulate that such patients may be at an increased risk of developing TNs. Consequently, we conducted a retrospective study to investigate the association between TNs and SII/AISI levels in T2DM patients, aiming to determine the predictive value of SII and AISI in identifying TNs.

## Methods

### Study design and population

**Fig. 1:**
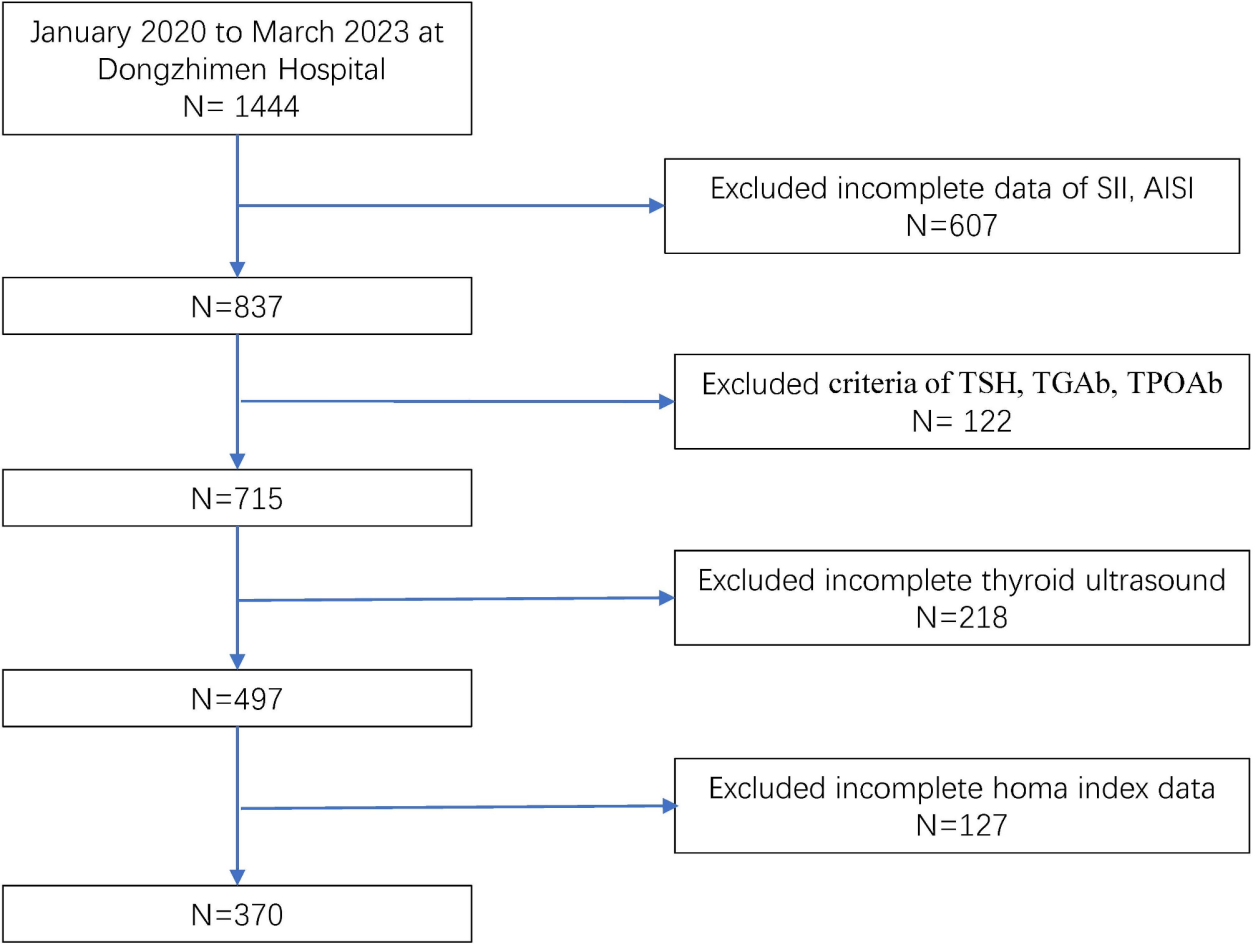
Flowchart of the participants selection from dongzhimen hospital

This retrospective study was conducted with the approval of the medical ethics committee at Dongzhimen Hospital, Beijing University of Chinese Medicine. Informed consent was not obtained since the study involved the utilization of data collected from inpatient electronic records. Eligible participants were identified among individuals aged 18 years or older, who had been diagnosed with type 2 diabetes mellitus (T2DM) according to the guidelines for prevention and treatment of type 2 diabetes in China (2020) [9], within the period from January 2020 to March 2023 at Dongzhimen Hospital (Fig. 1). Additionally, participants were required to have a confirmed diagnosis of diabetic kidney disease (DKD) in accordance with the Chinese guidelines for the diagnosis and treatment of DKD [10]. Exclusion criteria encompassed incomplete clinical data, a history of thyroid surgery, previous use of levothyroxine tablets or other antithyroid drugs, severe liver disease, infection, autoimmune disease, hematologic diseases, neoplasms, acute diabetic complications, hypoglycemia, and acid-base disturbances within the preceding 2 weeks. Furthermore, participants were included in the study if their Thyroid-Stimulating Hormone (TSH) levels fell within the normal range, and the levels of Thyroid Peroxidase Antibody (TPO) and Thyroglobulin Antibody (TGAB) were within 10 times the upper limit of their respective normal values.

### Laboratory assays

Biochemical parameters, including glycated hemoglobin type A1c (HbA1c, %), fasting plasma glucose (FPG, mmol/L), triglycerides (TG, mmol/L), total cholesterol (CHO, mmol/L), serum creatinine (Scr, µmol/L), blood urea nitrogen (BUN, mmol/L), uric acid (UA, µmol/L), High-density lipoprotein cholesterol (HDL, mmol/L), and Low-density lipoprotein cholesterol (LDL, mmol/L), were extracted from participants’ records. Assessment of thyroid function included free FT4 (reference range 5.44–11.85 µg/dL), free FT3 (reference range 0.66–1.61 ng/mL), serum thyroid stimulating hormone (TSH, reference range 0.56–5.91 uIU/mL), TT4 (reference range 5.44–11.85 µg/dL), TT3 (reference range 0.66–1.61 ng/mL), Thyroid peroxidase antibody (TPOAb, reference range 0.0–9.0 IU/ml), and thyroglobulin antibody (TGAb, reference range 0.0–9.0 IU/ml). These values were obtained from existing data. The estimated glomerular filtration rate (eGFR) was calculated using the CKD-EPI formula [11]. Ultrasound reports provided the data on thyroid nodules (TNs). All ultrasound examinations were conducted by radiologists using a 6–15 MHz/50mm linear probe (Logiqe 9, GE Medical Systems, WI, USA). The systemic immune-inflammation index (SII) was calculated as the product of platelet count and neutrophil-to-lymphocyte ratio, while the aggregate index of systemic inflammation (AISI) was determined as the product of neutrophil count, platelet count, and monocyte-to-lymphocyte ratio. Individual HOMA-IR was computed as HOMA-IR = (fasting insulin × fasting glucose)/405 with glucose measured in mg/dL and insulin in µU/L.

### Statistical analysis

Continuous variables that followed a normal distribution were presented as mean ± standard deviation (SD). The independent samples t-test was employed to compare these variables. Skewed data (determined by the Kolmogorov–Smirnov test with a significance level of p < 0.1 for each variable) were expressed as median (interquartile range). The Mann–Whitney U-test was used to assess differences in clinical characteristics between groups. Categorical variables were analyzed using the χ2 test. The ability of SII and AISI to identify T2DM-TNs patients was evaluated through ROC curve analysis. Binary logistic regression models with multivariable adjustment for various factors, including age, duration of diabetes, diabetic nephropathy (DN), TT3, FT3, TSH, TT4, FT4, homeostatic model assessment of insulin resistance (HOMA-IR), fasting plasma glucose (FPG) and glycated hemoglobin type A1c (HbA1c) were used to determine the association between SII and AISI quartiles and TNs. Statistical analysis was performed using SPSS software (Statistical Package for the Social Sciences, version 25.0, Chicago). A p-value less than 0.05 was considered statistically significant.

## Results

The average age of the entire participant cohort was 62.0 years. The TNs group exhibited a higher average age of 65.0 years, which was significantly greater than the average age of 52.0 years observed in the non-TNs group (z = -6.353, p < 0.001). Among the total population, 56.8% were male. In the TNs group, the proportion of males was lower (45.9%), whereas the non-TNs group had a higher proportion of males (74.5%). Furthermore, the TNs group displayed higher SII and AISI compared to the non-TNs group [443.2(338.3) vs. 335.1(234.4), p < 0.001 and 173.2(180.7) vs. 134.9(86.7), p < 0.001 for SII and AISI, respectively]. These differences were statistically significant. (Table 1)

**Table 1:** Clinical data of diabetic subjects with and without TNs

Characteristics	Total	TNs	Non-TNs	z/t//χ2 values	P values
	N = 370	N = 229	N = 141		
Age(y)	62.0(21.0)	65.0(16.0)	52.0(23.0)	-6.353	< 0.001
Gender, male (%)	210(56.8)	105(45.9)	105(74.5)	29.117	< 0.001
Duration of diabetes(y)	10.0(15.0)	13.0(14.0)	7.0(14.0)	-3.887	< 0.001
DN, n (%)	167(45.1)	116(50.7%)	51(36.2%)	7.394	0.007
SII	400.0(297.3)	443.2(338.3)	335.1(234.4)	-4.677	< 0.001
AISI	151.1(141.9)	173.2(180.7)	134.9(86.7)	-4.314	< 0.001
TT3(ng/ml)	0.8(0.3)	0.8(0.2)	0.9(0.3)	-2.297	0.017
FT3 (pg/mL)	3.0(1.5)	3.0(0.6)	3.1(0.6)	-2.720	0.007
TSH (µIU/ml)	1.8(1.4)	1.8(1.5)	1.8(0.9)	-0.766	0.444
TT4 (µg/dL)	8.9 ± 1.7	9.0 ± 1.7	8.6 ± 1.7	2.253	0.025
FT4 (ng/dL)	0.9(0.2)	0.9(0.2)	0.9(0.2)	-0.809	0.419
TPOAB (IU/ml)	0.5(0.8)	0.5(0.9)	0.5(0.8)	-0.731	0.465
TGAB (IU/ml)	0.0(0.1)	0.0(0.2)	0.0(0.1)	-0.415	0.678
Scr (µmol/L)	66.0(29.0)	65.0(31.0)	66.0(26.0)	-0.090	0.928
eGFR(ml/min)	94.4(27.6)	91.3(30.8)	104.5(25.2)	-4.909	< 0.001
BUN (mmol/L)	5.4(2.6)	5.6(3.0)	5.1(2.7)	-2.194	0.028
UA (µmol/L)	321.5(149.0)	325.0(154.0)	312.0(130.0)	-1.360	0.174
FPG (mmol/L)	8.0(4.2)	8.2(4.5)	7.7(3.8)	-1.564	0.118
HbA1c (%)	8.9(3.2)	8.7(3.3)	9.3(3.1)	-1.568	0.117
HOMA-IR	4.0(4.5)	4.2(5.0)	3.6(3.8)	-1.768	0.077
INS (mIU/L)	11.4(10.3)	11.6(10.8)	10.7(10.1)	-1.286	0.198
C-P(ng/ml)	2.0(1.7)	2.0(1.7)	2.0(1.8)	-0.259	0.795
TG (mmol/L)	1.6(1.2)	1.7(1.3)	1.6(1.0)	-0.996	0.319
CHO (mmol/L)	4.3(1.5)	4.4(1.5)	4.3(1.5)	-0.882	0.378
HDL (mmol/L)	1.0(0.4)	1.0(0.4)	0.9(0.3)	-1.551	0.121
LDL (mmol/L)	2.6 ± 0.9	2.6 ± 0.9	2.6 ± 0.9	-0.039	0.969

Note: Continuous variables were reported as mean ± standard deviation (SD) for normalized distribution or median with interquartile range (IQR) for non-normal variables, and categorical variables were reported as n (%)

Abbreviations: SII, systemic immune-inflammation index; AISI, aggregate index of systemic inflammation; TT3, total Triiodothyronine; FT3, free triiodothyronine; TSH, thyroid stimulating hormone; TT4, total thyroxine; FT4, free thyroxine; TPOAB, Thyroid peroxidase antibody; TGAB, thyroglobulin antibody; Scr, serum creatinine; eGFR, estimated glomerular filtration rate; BUN, blood urea nitrogen; UA, uric acid; FPG, fasting plasma glucose; HbA1c (%), glycated hemoglobin type A1c; INS, insulin; C-P, C-peptide; TG, triglycerides; CHO, total cholesterol; HDL, high-density lipoprotein cholesterol; LDL, low-density lipoprotein cholesterol; HOMA-IR, (fasting insulin × fasting glucose)/405 with glucose measured in mg/dL and insulin in µU/L.

Age, duration of diabetes, diabetic nephropathy (DN), systemic immune-inflammation index (SII), and aggregate index of systemic inflammation (AISI) exhibit significant positive associations with thyroid nodules (TNs). However, other variables such as total triiodothyronine (TT3), free triiodothyronine (FT3), thyroid-stimulating hormone (TSH), total thyroxine (TT4), free thyroxine (FT4), estimated glomerular filtration rate (eGFR), serum creatinine (Scr), blood urea nitrogen (BUN), and homeostatic model assessment of insulin resistance (Homa-ir) do not demonstrate significant associations with TNs. When stratifying the data by gender, significant associations observed in males, including age, duration of diabetes, DN, SII, AISI, FT3, and eGFR, are consistent with those observed in the total participant cohort. However, in females, SII and AISI do not exhibit significant associations with TNs. (Table 2)

**Table 2:** Univariate binary logistic regression for thyroid nodules

	Total		Male		Female	P values
	OR (95%CI)	P values	OR (95%CI)	P values	OR (95%CI)	
Age(y)	1.057(1.039 ~ 1.076)	< 0.001	1.036(1.014 ~ 1.058)	0.001	1.075(1.039 ~ 1.111)	< 0.001
Duration of diabetes(y)	1.049(1.022 ~ 1.076)	< 0.001	1.030(0.997 ~ 1.064)	0.073	1.054(1.006 ~ 1.105)	0.027
DN, n (%)	1.812()1.178 ~ 2.786	0.007	2.642(1.513 ~ 4.612)	0.001	1.444(0.663 ~ 3.149)	0.355
SII	1.002(1.001 ~ 1.003)	< 0.001	1.003(1.002 ~ 1.005)	< 0.001	1.001(1.000 ~ 1.002)	0.226
AISI	1.004()1.002 ~ 1.006	< 0.001	1.006(1.003 ~ 1.010)	< 0.001	1.002(0.999 ~ 1.004)	0.127
TT3(ng/ml)	0.272(0.085 ~ 0.872)	0.029	0.251(0.058 ~ 1.091)	0.065	0.574(0.067 ~ 4.906)	0.612
FT3 (pg/mL)	0.518(0.334 ~ 0.804)	0.003	0.607(0.349 ~ 1.058)	0.078	0.605(0.274 ~ 1.339)	0.215
TSH (uIU/ml)	1.113(0.915 ~ 1.353)	0.284	1.091(0.835 ~ 1.426)	0.521	0.956(0.700 ~ 1.307)	0.778
TT4 (ug/dL)	1.158(1.018 ~ 1.318)	0.026	1.127(0.949 ~ 1.339)	0.174	1.071(0.861 ~ 1.332)	0.538
FT4 (ng/dL)	0.572(0.138 ~ 2.379)	0.443	0.335(0.055 ~ 2.032)	0.235	2.681(0.181 ~ 39.797)	0.474
eGFR(ml/min)	0981(0.973 ~ 0.990)	< 0.001	0.983(0.973 ~ 0.993)	0.001	0.980(0.964 ~ 0.996)	0.014
Scr (umol/L)	1.003(1.000 ~ 1.006)	0.073	1.006(1.000 ~ 1.012)	0.040	1.002(0.997 ~ 1.006)	0.528
BUN (mmol/L)	1.058(1.000 ~ 1.120)	0.050	1.078(0.999 ~ 1.164)	0.053	1.034(0.943 ~ 1.133)	0.483
HOMA-IR	1.009(0.994 ~ 1.025)	0.259	1.009(0.992 ~ 1.027)	0.296	1.009(0.977 ~ 1.042)	0.582

Note: Variables were calculated using the univariate binary logistic regression model. The effect of each variable on thyroid nodules was presented in odds ratio and 95% confidence interval. Odds ratio greater than 1 represented increased odds for thyroid nodules. P values were calculated using univariate binary logistic regression analysis

Abbreviations: SII, systemic immune-inflammation index; AISI, aggregate index of systemic inflammation; TT3, total Triiodothyronine; FT3, free triiodothyronine; TSH, thyroid stimulating hormone; TT4, total thyroxine; FT4, free thyroxine; Scr, serum creatinine; eGFR, estimated glomerular filtration rate; BUN, blood urea nitrogen; HOMA-IR, (fasting insulin × fasting glucose)/405 with glucose measured in mg/dL and insulin in µU/L.

In the total participants and among males, there is an observed increase in the risk of thyroid nodules (TNs) as the systemic immune-inflammation index (SII) quartiles progress from the first to the fourth quartile. Hazard ratios (HRs) for TNs were 1.578 (0.883 ~ 2.820), 2.279 (1.257 ~ 4.131), and 3.626 (1.931 ~ 6.810) for the second, third, and fourth quartiles of SII, respectively. The p-values for trend indicate a statistically significant trend, with a value of P < 0.001. These associations remain significant even after adjusting for confounding factors. However, among females, the associations between SII quartiles and TNs are not statistically significant. Similar results are observed for the aggregate index of systemic inflammation (AISI) and its association with TNs. Please refer to Tables 3 and 4 for further details.

**Table 3:** Association between SII and TNs in all participants and stratified by gender

	Quartiles 1	Quartiles 2	Quartiles 3	Quartiles 4	P for trend
Total	231.8	343.6	480.3	781.0	
Cases/participants	43/92	54/93	62/93	70/92	
Model 1	1.0	1.578(0.883 ~ 2.820)	2.279(1.257 ~ 4.131)	3.626(1.931 ~ 6.810)	< 0.001
Model 2	1.0	1.373(0.726 ~ 2.594)	2.055(1.072 ~ 3.938)	2.415(1.195 ~ 4.880)	0.011
Model 3	1.0	1.366(0.723 ~ 2.579)	2.144(1.119 ~ 4.108)	2.457(1.212 ~ 4.983)	0.009
Men	236.1	328.3	440.2	696.8	
Cases/participants	15/52	27/53	29/53	34/52	
Model 1	1.0	2.562(1.144 ~ 5.736)	2.981(1.329 ~ 6.685)	4.659(2.035 ~ 10.669)	0.001
Model 2	1.0	2.195(0.913·5.278)	2.600(1.088 ~ 6.212)	3.518(1.420 ~ 8.716)	0.013
Model 3	1.0	1.900(0.781 ~ 4.619)	2.840(1.180 ~ 6.833)	3.861(1.526 ~ 9.770)	0.006
Women	226.7	370.1	561.9	975.8	
Cases/participants	27/40	31/40	35/40	31/40	
Model 1	1.0	1.658(0.614 ~ 4.482)	3.370(1.070 ~ 10.613)	1.658(0.614 ~ 4.482)	0.369
Model 2	1.0	1.259(0.409 ~ 3.879)	3.617(0.930 ~ 14.066)	0.974(0.285 ~ 3.325)	0.970
Model 3	1.0	1.107(0.346 ~ 3.539)	3.701(0.924 ~ 14.820)	0.881(0.245 ~ 3.174)	0.969

Model 1, unadjusted

Model 2, adjusted for age, duration of diabetes, DN, TT3, FT3, TSH, TT4, FT4

Model 3, further adjusted for Homa-ir, FPG, HbA1c based on model 2

**Table 4:** Association between AISI and TNs in all participants and stratified by gender

	Quartiles 1	Quartiles 2	Quartiles 3	Quartiles 4	P for trend
Total	69.7	128.9	188.7	358.6	
Cases/participants	45/92	52/93	60/93	72/92	
Model 1	1.0	1.325(0.743 ~ 2.362)	1.899(1.053 ~ 3.424)	3.760(1.978 ~ 7.147)	< 0.001
Model 2	1.0	1.589(0.839 ~ 3.008)	1.720(0.895 ~ 3.305)	3.257(1.602 ~ 6.622)	0.001
Model 3	1.0	1.594(0.839 ~ 3.028)	1.714(0.889 ~ 3.306)	3.238(1.584 ~ 6.617)	0.002
Men	77.1	126.1	178.1	317.7	
Cases/participants	15/52	25/53	27/53	38/52	
Model 1	1.0	2.202(0.983 ~ 4.934)	2.562(1.144 ~ 5.736)	6.695(2.840 ~ 15.783)	< 0.001
Model 2	1.0	2.298(0.953 ~ 5.539)	2.442(1.016 ~ 5.868)	6.884(2.702 ~ 17.542)	< 0.001
Model 3	1.0	2.530(1.023 ~ 6.260)	2.473(0.999 ~ 6.125)	8.573(3.175 ~ 23.148)	< 0.001
Women	64.9	132.4	204.1	430.6	
Cases/participants	30/41	18/25	34/43	42/51	
Model 1	1.0	0.879(0.324 ~ 2.381)	1.148(0.409 ~ 3.219)	1.889(0.613 ~ 5.818)	0.189
Model 2	1.0	1.037(0.334 ~ 3.219)	1.095(0.341 ~ 3.518)	1.440(0.399 ~ 5.196)	0.549
Model 3	1.0	0.901(0.280 ~ 2.900)	1.043(0.310 ~ 3.506)	1.356(0.360 ~ 5.111)	0.572

Model 1, unadjusted

Model 2, adjusted for age, duration of diabetes, DN, TT3, FT3, TSH, TT4, FT4

Model 3, further adjusted for Homa-ir, FPG, HbA1c based on model 2

**Fig. 2:**
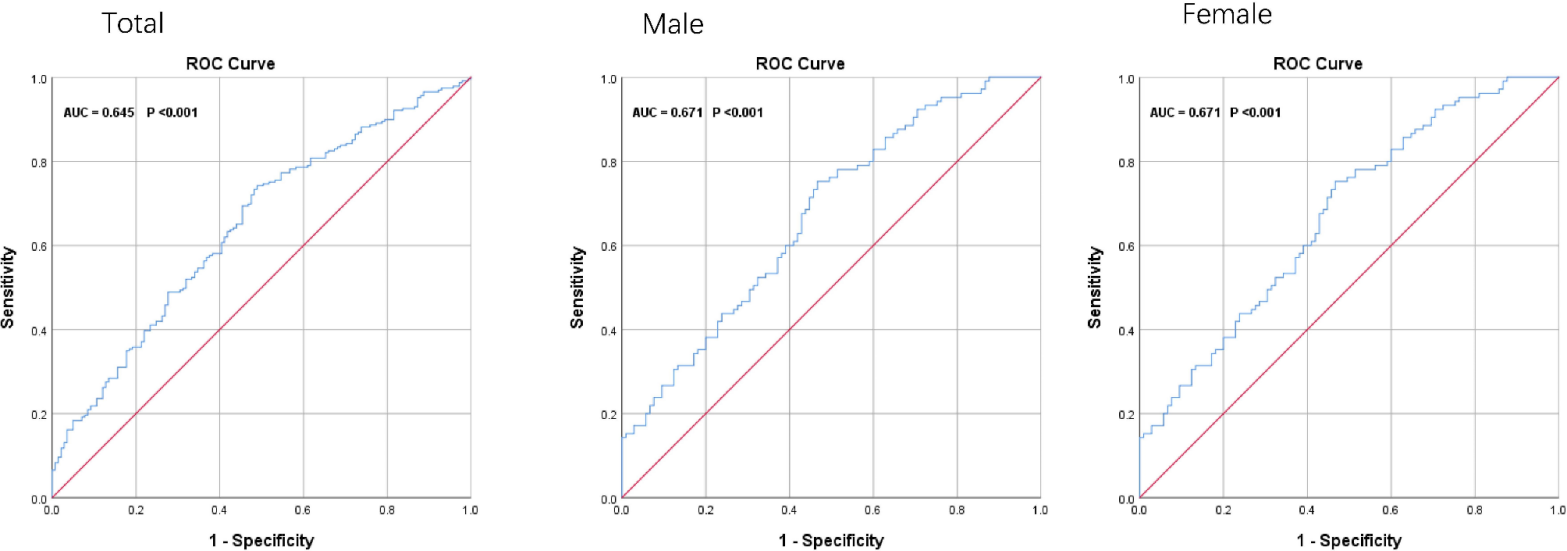
ROC Curve analysis of the ability of SII to predict T2DM-TN and stratified by gender

**Fig. 3:**
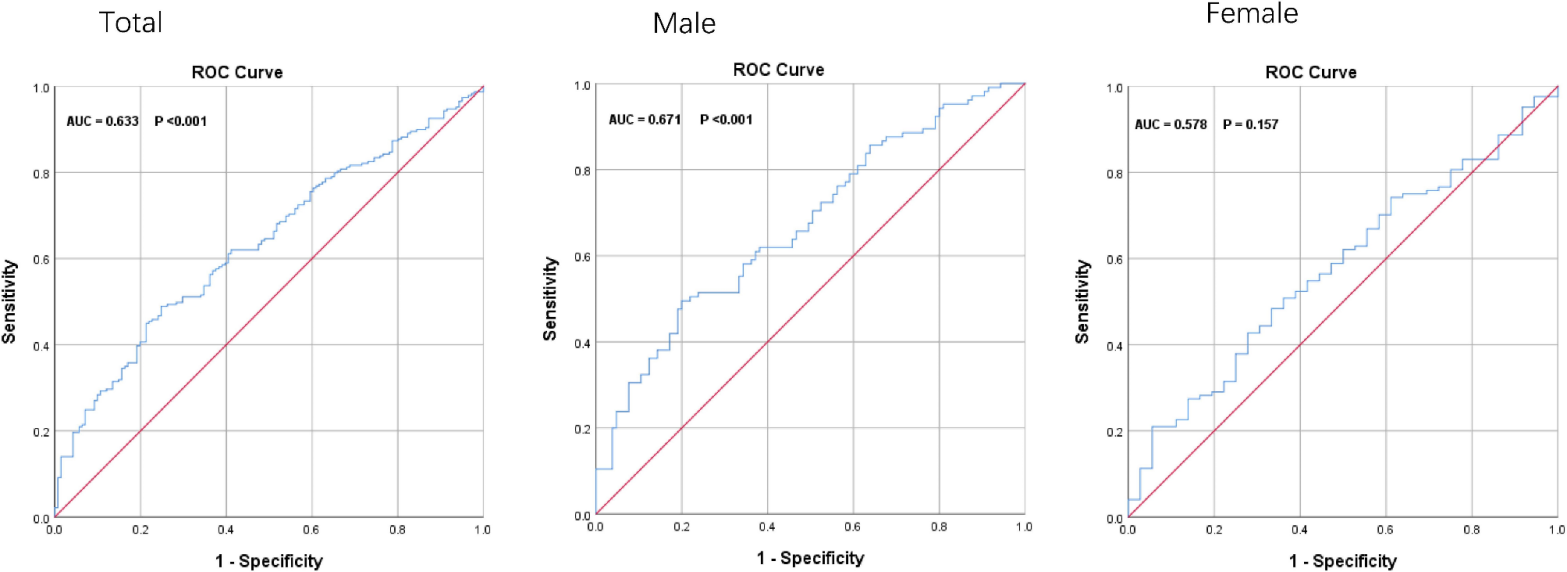
ROC Curve analysis of the ability of AISI to predict T2DM-TN and stratified by gender

The ROC curve analysis was used to evaluate the ability of SII, AISI to identify T2DM-TNs patients. The results of the ROC curve showed that SII and AISI exhibited a high discriminating value for TNs in total participants and male. There were no significant in female. (Figures 2 and 3)

## Discussion

In this retrospective study involving 370 patients with type 2 diabetes mellitus (T2DM), we observed a prevalence of thyroid nodules (TNs) of 61.9%, which is consistent with findings reported by Zhang [1]. Our study revealed an association between the systemic immune-inflammation index (SII) and aggregate index of systemic inflammation (AISI) quartiles with the presence of thyroid nodules. Importantly, this association remained statistically significant even after adjusting for potential confounding factors. Upon further analysis stratified by gender, we observed that this association was only present among male patients, while no significant association was found among female patients. To the best of our knowledge, this is the first study to demonstrate the association between SII, AISI, and TNs specifically in patients with T2DM. Our findings indicate that SII and AISI levels were higher in T2DM patients with thyroid nodules compared to those without, highlighting the potential role of systemic inflammation in the development of thyroid nodules in male T2DM patients. However, it is important to note that no significant association was observed in female patients. Type 2 diabetes mellitus (T2DM) is unequivocally established as an inflammatory condition, characterized by perturbations in various inflammatory mediators, notably tumor necrosis factor-α (TNFα), interleukin-1β (IL-1β), and interleukin-6 (IL-6), which have been strongly associated with the development of diabetic complications [12, 13]. Our investigation underscores the intricate interplay between systemic inflammation and endocrine-metabolic dysregulation, particularly in the context of T2DM. Furthermore, it is noteworthy that the systemic immune-inflammation index (SII) and aggregate index of systemic inflammation (AISI) represent clinical biomarkers that offer distinct advantages in terms of accessibility and convenience for routine patient monitoring when compared to more elaborate inflammatory markers.

SII and AISI represent biomarkers that encompass multiple peripheral inflammatory cells in the body, including neutrophils, lymphocytes, platelet counts, monocytes, and other parameters associated with the inflammatory response. These composite indices provide a comprehensive assessment of systemic inflammatory activity and offer advantages over individual inflammatory markers in terms of ease of detection. The concept of SII was initially introduced by HU [14] in a study involving 2006 patients with liver cancer and has since demonstrated prognostic value in various malignancies such as colorectal cancer, hepatocellular carcinoma, and lung cancer [5, 15, 16]. More recently, SII has garnered attention in different fields, including endocrinology. For instance, Wang [17] observed significantly higher SII levels in individuals with type 2 diabetes mellitus (T2DM) and comorbid depression compared to those without depression, highlighting SII as an independent risk factor for depression in T2DM patients. Similarly, AISI has shown promise in distinguishing patients with idiopathic pulmonary fibrosis (IPF), with AISI levels being independently associated with poor prognosis in this population [18]. Notably, Song [8] identified a robust association between SII, AISI, and an increased prevalence of peripheral arterial disease in T2DM patients, suggesting a relationship with disease severity. Monocytes, recognized as the vanguard of infiltrating cells in the pathogenesis of diabetic complications, hold a pivotal role in the genesis of these complications [19, 20]. Investigations have substantiated that elevations in circulating inflammatory markers are intimately linked to the extent of retinal damage observed in diabetic patients [21]. In the milieu of Type 2 Diabetes Mellitus (T2DM), the Systemic Immune-Inflammation Index (SII) and Aggregate Index of Systemic Inflammation (AISI) have emerged as novel indicators of circulating inflammatory activity, encompassing a diverse array of peripheral inflammatory cells and mediators. This elevation in SII and AISI is emblematic of a persistent inflammatory cascade, akin in its deleterious consequences to other pro-inflammatory factors. This chronic state of inflammation imperils the intricate machinery of insulin signaling, fosters insulin resistance, and thereby precipitates the onset of diabetic complications. Notably, extant studies have underscored the nexus between inflammatory processes and the emergence of diabetic complications, such as diabetic nephropathy and diabetic retinopathy. Consequently, vigilant monitoring for aberrations in SII and AISI in diabetic individuals could potentially herald the need for timely intervention with anti-inflammatory therapeutics, offering promise in mitigating the initiation and progression of diabetic complications.

This study found that SII and AISI were independent risk factors for TNs in patients with T2DM, and high levels of SII and AISI could promote the occurrence of TNs. With the spread of ultrasonography, the prevalence of TNs has gradually increased, and its etiology is diverse. Inflammation can directly damage thyroid tissue, cause thyroid inflammation, and further promote thyroid tissue and cell proliferation [22]. Studies have shown that TNs are closely related to inflammatory factors. Sabahattin [23] found that the amount of TNs was positively correlated with CRP and negatively correlated with fibrinogen levels. Cao [24] found that MAU is an independent risk factor for TNs, and in T2DM, inflammatory damage can promote kidney and thyroid damage, and induce the occurrence of MAU and TNs. Li [25] believes that inflammation is the main mechanism that promotes the occurrence and development of TNs disease, and may play an indirect role in inhibiting the synthesis of thyroid hormones through inflammation. SII and AISI as inflammatory indicators may regulate the activity of inflammatory cells in a variety of ways, and studies have found that the elevation of SII and AISI is associated with the increase of pro-inflammatory factors (e.g., tumor necrosis factor-alpha, interleukin-6, etc.) and inflammatory mediators [26, 27], thereby promoting the occurrence and development of thyroid nodules. However, the specific mechanism of action needs further research to explain. Understanding the biological significance and underlying mechanisms of SII and AISI in thyroid nodules can help identify potential therapeutic targets and provide individualized treatment for the prevention and management of thyroid nodules in diabetic patients.

In this investigation, we observed a higher prevalence of thyroid nodules in women, and the occurrence of thyroid nodules demonstrated an upward trend with longer diabetes duration and older age, consistent with our previous findings [24]. Notably, our study revealed that elevated SII and AISI levels were independent risk factors for the development of thyroid nodules in male diabetic patients. Previous research has indicated that men exhibit significantly higher levels of inflammatory markers such as IL-1β, IL-6, and TNF-α compared to women, suggesting a gender-dependent influence on inflammatory factor production, potentially mediated by testosterone and other factors [28]. The present study unveiled a strong association between SII, AISI, and thyroid nodule formation, particularly in male patients, while this association was not observed in female patients. It is worth noting that the sample size of female participants in our study may have affected the statistical assessment. Therefore, further research and validation are necessary to determine the precise magnitude of this gender-related phenomenon. Overall, our findings suggest that SII and AISI hold promise as independent risk factors for thyroid nodules, serving as potential screening and predictive markers for thyroid disease risk in individuals with diabetes. However, additional studies and clinical trials are warranted to confirm the feasibility and efficacy of their clinical application.

Owing to the limitation in sample size, the current study acknowledges the potential impact on result stability and reliability. Therefore, it is recommended to undertake future investigations with a larger sample size to enhance the robustness of the findings. Furthermore, this study utilized a retrospective design, warranting additional prospective long-term follow-up observations to better elucidate the relationship between SII and AISI with thyroid nodules and ascertain their long-term predictive value as biomarkers. In terms of inflammatory markers, this study exclusively focused on SII and AISI, overlooking other potentially relevant indicators. To obtain a more comprehensive evaluation of inflammatory status and gain a deeper mechanistic understanding, a more extensive analysis of inflammatory markers could be employed. Additionally, the current study predominantly centered on male patients with type 2 diabetes, with the results failing to exhibit statistically significant association among female patients. Thus, future investigations should emphasize exploring the association between SII, AISI, and thyroid nodules in patients of varying genders, while elucidating the gender-specific pathological and physiological mechanisms involved.

## Conclusions

This study provides evidence that SII and AISI are independent risk factors for TNs in individuals with T2DM, suggesting that elevated SII and AISI levels may contribute to the development of TNs.

## Data Availability

The datasets generated and/or analyzed during the current study are not publicly available due to privacy and ethical considerations, but can be available from the corresponding author on reasonable request.
